# Level of Adherence to COVID-19 Preventive Measures Among Health Care Workers in Saudi Arabia

**DOI:** 10.7759/cureus.15969

**Published:** 2021-06-27

**Authors:** Fatimah I Albeladi, Maryam M Alluli, Khaled A Daghriri, Yahya H Almalki, Mousa Y Wafi, Faisal A Otaif, Zahra Y Sulays, Amro A Hakami, Ahmad A Alharbi, Abdulaziz H Alhazmi

**Affiliations:** 1 College of Medicine, Jazan University, Jazan, SAU

**Keywords:** covid-19, prevention, saudi arabia, adherence, sars-cov-2

## Abstract

Background: Coronavirus disease 2019 (COVID-19) is a pandemic caused by a virus called severe acute respiratory syndrome coronavirus 2 (SARS-CoV-2). Health-care workers (HCWs) are at a high risk of contracting SARS-CoV-2 infection. Thus, different infection control strategies have been used to reduce SARS-CoV-2 transmission. Our study aims to assess the level of adherence of HCWs to the preventive measures against COVID-19 in Saudi Arabia.

Methods: An observational study was carried out using data collected by a self-administrated dual-language (Arabic and English) online questionnaire directed to HCWs in Saudi Arabia to measure the level of adherence to COVID-19 preventive measures. All HCWs involved in patient care in Saudi Arabia were included in this study.

Results: A total of 214 HCWs were included in the study (median age = 30 years; 62% male). Among all the participants, 65% of HCWs were in direct contact with COVID-19 patients, and 18% were diagnosed with COVID-19. The level of overall adherence to mask use was 82%. HCWs were committed to wearing gloves, gowns, and goggles with a percent of 95%, 85%, and 68%, respectively.

Conclusion: Our findings demonstrated that HCWs in Saudi Arabia have an acceptable level of adherence to COVID-19 preventive measures during the pandemic. Larger studies are required to evaluate the effectiveness of these preventive measures in reducing the transmission of respiratory microbes between HCWs and patients.

## Introduction

Early in 2020, the World Health Organization (WHO) declared coronavirus disease 2019 (COVID-19) as a pandemic [1]. It is an illness, with no specific treatment, that rapidly transmitted across the globe and made most of the health officials concerned about its rate of transmission, clinical burden, and outcomes [2].

Health-care workers (HCWs) are the front-line fighters in this pandemic, and they are at a higher risk of COVID-19 infection due to their proximity to COVID-19 patients. Thus, they need to ensure their safety and protection from contracting infectious agents due to high exposure to these microbes at the hospital [2]. There are many infection control strategies, which can be named as administrative control measures, environmental control measures, and the use of personal protective equipment (PPE) [3]. PPE is used in healthcare settings as a standard-based precaution to protect HCWs from infections and prevent further spread of the microbes to the patients [3]. PPE is ranked as the lowest in the infection control hierarchy and is recommended by most infection control guidelines to be used with the other strategies to ensure higher efficiency and protection [3]. However, PPE is used during the early stages of an outbreak or pandemic, especially when there are no effective treatments or vaccines [3]. Several non-pharmaceutical interventions (NPIs) have proven their utility in preventing microbes' transmission, such as hand washing, cough etiquette, mask-wearing, physical distancing, and isolation [4]. The importance of proper application of the NPIs is further stressed in the elderly populations and individuals with comorbidities who may suffer from serious outcomes of COVID-19. However, most infected individuals (who may remain asymptomatic or experience mild manifestations) can transmit the virus, and thus infection control measures should not be neglected [4]. In India, one study showed that there is a reduction in COVID-19 cases by almost 90% due to the use of preventive measures [4]. Moreover, Canada reported that the physical distance of 1 meter or more, using face masks and eye protection, resulted in a large reduction in the risk of infection among healthcare and non-healthcare workers [5]. In Saudi Arabia, many unprecedented and stringent preventive and precautionary measures have been taken to control COVID-19, such as banning international and domestic flights, closing mosques, schools, and restaurants [6]. Moreover, various recommendations of preventive measures have been directed toward HCWs as well as the general population [7]. However, there is little information on the level of adherence of HCWs in Saudi Arabia to the COVID-19 prevention measures. Thus, this study aims to evaluate the level of adherence of HCWs to preventive measures during the COVID-19 pandemic.

## Materials and methods

This study is an observational, descriptive, web-based, dual language (Arabic and English) survey to measure the level of adherence of HCWs to the COVID-19 prevention measures. It is a one-month-long study conducted between September 19 and October 19, 2020. HCWs who are involved in patient care in Saudi Arabia were included in this study. Individuals who refused to complete the online survey and non-healthcare workers were excluded. The data was collected as a self-administrated online questionnaire. The questionnaire comprised two parts: the first part covered the socio-demographic details of participants such as age, gender, nationality, residence, hospital, job, etc., and the second part included 10 close-ended questions to measure the level of adherence to the preventive measures. The representative target sample size needed to achieve sufficient statistical power was 267 participants, using a margin of error of ±5%, a confidence level of 90%, a 50% response distribution, and 20,000 people. The collected data were verified manually, entered into a personal computer, and then analyzed using SPSS Statistics, version 22.0 (IBM Corp., Armonk, NY). Descriptive statistics were calculated for study variables such as frequency and percentage for qualitative variables and mean and standard deviation for quantitative variables. Appropriate significance tests (including t-test and chi-square) were applied, and p < 0.05 was considered to be statistically significant. The study had been ethically approved by Jazan Health Ethics Committee (approval number 2039, dated 28/8/2020). Informed consent was obtained from all participants.

## Results

A total of 214 HCWs responded to the questionnaire and were included in our study. The median age of participants was 30, with a range of 19 to 62 years (Table 1). Most participants, a total of 133 (62%), were male. The majority of HCWs were doctors, a total of 112 (52%), and others were as following: 35 (16%) nurses, 19 (8.9%) technicians, 2 (0.9%) respiratory therapists, 1 (0.5%) paramedic, and 45 other health-care workers (including social workers, security personnel, health educationists, physical therapists, dentists, pharmacists, dietitians, and health informatics). Most of the participants were from Jazan 106 (49.5%), followed by Dammam 31 (14.5%), then Riyadh 31 (14.5%), Makkah 19 (8.90%), Asir 16 (7.5%), Najran 4 (1.90%), Al-Jouf 2 (0.90 %), Al-Baha 2 (0.90%), Tabouk 2 (0.50%), Madina 1 (0.50%), and Al-Qassim 1 (0.50%). HCWs who were in direct contact with COVID-19 patients were 65% (139), and 18% (39) were diagnosed with COVID-19. The level of adherence among HCWs in Saudi Arabia to the preventive measures during the COVID-19 pandemic is summarized in Table 1.

**Table 1:** General characteristics of the participants and the level of adherence to the preventive measures among HCWs in Saudi Arabia HCWs: Health-care workers. SD: Standard deviation.

Characteristics	HCWs (n=214)
Median age in years (SD; range)	30 (9; 19 to 62)
Male	133 (62%)
Mask use	205 (94%)
Mask reuse	73 (34%)
Surgical mask use	177 (83%)
N95 mask use	21 (10%)
Cotton mask use	16 (8%)
Environmental cleaning and disinfection	180 (84%)
Wearing goggles or a face shield	146 (68%)
Wearing gown	182 (85%)
Gloves use	203 (95%)

In Table 2, we present a comparison between doctors and other healthcare practitioners in the level of adherence to the preventive measures during the COVID-19 pandemic. The mask reuse tendency was significantly higher among doctors (40%) compared to non-doctors (27%), also cleaning and disinfection of the patient’s environment were more significantly practiced among non-doctors (92%) compared to the doctors (76%). No significant difference was noted in all other preventive measures, such as wearing goggles, gowns, and gloves. 

**Table 2:** The level of adherence to the preventive measures among doctors and other health practitioners. SD: Standard deviation * statistically significant

Study Parameters	Doctors	Non-doctors	p-value
Number (%)	112 (55%)	102 (45%)	0.144
Age; median (SD)	31 (10)	30 (8)	0.402
Male (%)	77	46	0.0001
Mask reuse	45 (40%)	28 (27%)	0.045*
Environmental cleaning	85 (76%)	94 (92%)	0.0016*
Wearing goggles	74 (66%)	72 (71%)	0.43
Wearing gown	95 (85%)	87 (85%)	1
Wearing gloves	108 (96%)	94 (92%)	0.21

## Discussion

SARS-CoV-2 is a highly transmissible virus, and it is the responsible agent for COVID-19 [5]. HCWs are at a high risk of contracting this virus due to their high exposure to infected individuals. Consequently, different recommendations for the preventive measure have been made by the official health authorities in Saudi Arabia to the general population and HCWs [7]. Some local and national studies were conducted in Saudi Arabia to evaluate the level of adherence to preventive measures against COVID-19, and these studies were carried out on the general population. For example, one study was conducted on the population of Jeddah city, and it was found that 49% (231) of the participants were adherent to COVID-19 preventive measures. Moreover, wearing a mask and hand washing were found to be the most followed preventive measures [8]. Another national study conducted during the early phase of the pandemic (between March and April 2020) found that hand washing was practiced among 64% of participants [9]. On the other hand, knowledge about COVID-19 among HCWs in Saudi Arabia was evaluated, and it was found that the majority of participants believed that social distancing, wearing face masks, and washing hands are effective methods for the prevention of disease transmission [10]. These findings are reflected in the current study, in which we aimed to evaluate the level of adherence of HCWs in Saudi Arabia to the recommended preventive measures against COVID-19. Our results showed that HCWs in Saudi Arabia have adhered well to preventive measures against COVID-19. More specifically, our results showed high levels of adherence to mask (96%), gown (85%), gloves (95%), goggles, and face shield (68%) wearing practices, along with adhering to washing hands (80%), and cleaning and disinfection of the patient’s environment (84%) practices.

At the international level, the compliance of HCWs to the infection control guidelines and preventive measures varied in general; however, it was enhanced during this pandemic. Our results showed that 80% of the participants wash hands before dealing with patients; while in Ethiopia, it is reported in a study conducted in 2014 that only 18% of HCWs wash hands before dealing with a patient, 40% before aseptic techniques, and 81% after removal of gloves [11]. In Jamaica, a study conducted between March and May 2009 showed that HCWs are highly adherent to the preventive measures, in which 87% of physicians and 88% of nurses perform handwashing when they are exposed to the body fluid [12]. Wearing a mask is another practice that indicates the effectiveness of reinforcement of infection control protocols during the pandemic to control the SARS-CoV-2 pandemic. For example, in Italy in 2010, only 35% of HCWs wore a mask [13], and only 18% of HCWs wore a mask according to another study conducted in 2007 in England [14]. In the current study, 82% of HCWs in Saudi Arabia always wore a mask, a finding that supports the idea that the HCWs in Saudi Arabia may have gained awareness and knowledge about COVID-19 and its transmission and understand the importance of infection control practices during the pandemic [10].

It is generally accepted that nurses have better knowledge and are more adherent to infection control practices, compared to doctors [15]. Thus, it is not surprising that doctors in our study group are less adherent to some infection control practices, such as reusing a mask and not cleaning and disinfecting the patient’s environment and surfaces, compared to other health practitioners. However, this gap of knowledge and practice must be reduced, and more efforts by doctors may be made to ensure better adherence to preventive measures and practices, especially during pandemics.

Despite it being one of the few studies in Saudi Arabia that discussed the level of adherence of HCWs to preventive measures, our study has many limitations. The data have been collected by a self-administered online questionnaire, and this could result in a reporting bias and may measure the HCWs' knowledge rather than the real practice. Due to the COVID-19 pandemic and the lockdown in Saudi Arabia, we preferred to conduct this study as a web-based study to ensure the safety of all study participants. Moreover, community-based surveys were not possible to be done during this pandemic. Therefore, data collection was online as a self-reported survey, and the distribution of this survey relied on the authors’ networks. As such, the majority of the participants (50%) were from Jazan province, where all of the authors come from. Further research should cover the perceptions of all regions of the country and include more HCWs from different categories.

## Conclusions

HCWs are front-line workers in the COVID-19 pandemic, a disease that is easily transmissible between individuals. In this study, we showed that HCWs in Saudi Arabia have an acceptable adherence level to the common preventive measure practices. Our result indicates that HCWs have gained knowledge and awareness about infection control practices. Further studies are needed on a larger population to evaluate the effectiveness of these measures in preventing or reducing microbe transmission between HCWs and patients.
